# Characterization of *Legionella pneumophila* Isolated from Environmental Water and Ashiyu Foot Spa

**DOI:** 10.1155/2013/514395

**Published:** 2013-07-11

**Authors:** Masato Tachibana, Masaya Nakamoto, Yui Kimura, Takashi Shimizu, Masahisa Watarai

**Affiliations:** ^1^The United Graduate School of Veterinary Science, Yamaguchi University, 1677-1 Yoshida, Yamaguchi 753-8515, Japan; ^2^Laboratory of Veterinary Public Health, Joint Faculty of Veterinary Medicine, Yamaguchi University, 1677-1 Yoshida, Yamaguchi 753-8515, Japan

## Abstract

Hot springs are the most common infectious source of *Legionella pneumophila* in Japan. However, little is known about the association between *L. pneumophila* and environmental waters other than hot springs. In this study, water samples from 22 environmental water sites were surveyed; of the 22 samples, five were *L. pneumophila* positive (23%). *L. pneumophila* was mainly isolated from ashiyu foot spas, a type of hot spring for the feet (3/8, 38%). These isolates had genetic loci or genes that encoded the virulence factors of *L. pneumophila*. Moreover, these isolates showed higher intracellular growth and stronger cytotoxicity compared with the reference strain. These results suggest that ashiyu foot spa can be the original source for *L. pneumophila* infection.

## 1. Introduction

*Legionella pneumophila* is the causative agent of legionellosis. In humans, *L. pneumophila* can induce Legionnaires' disease and Pontiac fever. Legionnaires' disease is a form of severe pneumonia, while Pontiac fever produces acute flu-like symptoms without pneumonia [1]. A number of factors including type II and type IV secretion systems, a pore-forming toxin, type IV pili, flagella, and heat shock proteins [2–7] contribute to *L. pneumophila* virulence. *L. pneumophila* is a facultative intracellular Gram-negative bacterium that can reside and multiply within free-living amoebae in environmental waters. *L. pneumophila* can withstand temperatures of 0–68°C and a pH range of 5.0–8.5 and survive in most environments for long periods [8]. *L. pneumophila* mainly lives in natural and man-made aquatic environment such as ponds, hot springs, fountains, cooling towers, and portable waters [8]. Hot springs and public baths are known to be most common source of *L. pneumophila* outbreaks in Japan [9–11]. Abundance information about the relationship between *L. pneumophila* and hot springs and public baths has been accumulated, but there is little information regarding *L. pneumophila* in environmental waters other than hot springs and public baths.

In this study, 22 environmental water places were surveyed in Yamaguchi Prefecture, Japan, and *L. pneumophila* was isolated from five sites.

## 2. Materials and Methods

### 2.1. Bacteria and Culture Conditions

*Legionella pneumophila* Lp02 and the *ΔdotA* mutant, Lp03 [2, 5], were maintained as frozen glycerol stocks and cultured on N-(2-acetamido-) 2-aminoethanesulphonic acid (ACES)-buffered charcoal-yeast extract broth containing 1.5% agar (CYET) or liquid ACES-buffered yeast extract broth (AYET) supplemented with 100 *μ*g/mL thymidine.

Isolation of *L. pneumophila* was performed using CYET supplemented with glycine (Wako, Osaka, Japan, 3 mg/mL), vancomycin HCl (Wako, 1 *μ*g/mL), polymyxin B (Sigma, MD, USA, 79.2 IU/mL), and sulfate cycloheximide (Wako, 80 *μ*g/mL) (GVPC agar) [12]. Isolated bacteria were grown on CYET at 37°C or in AYET with shaking [13].

### 2.2. Specimen Collection and Preparation

Samples were collected from 22 environmental water sites. Eight samples were collected from ashiyu foot spas, seven were from water fountains, four were from basins of shrine, and three were from ponds (Table 1). Five hundred milliliters of sample was collected from each site in sterile bottles or small plastic containers and centrifuged at 3000 rpm for 20 min at 4°C. The deposits were resuspended in 500 *μ*L distilled water as concentrates. Concentrated samples were heated at 50°C for 30 min and spread onto the surface of GVPC agar. Plates were incubated at 37°C and they were inspected daily.

### 2.3. PCR Analysis

The primers used for PCR analysis are summarized in Table 2. After denaturation of the bacterial chromosomal DNA template at 95°C for 5 min, 35 cycles of PCR amplification were performed using expand high fidelity PCR system (Roche, Basel, Switzerland). 

### 2.4. Serotyping

Serotypes of isolated bacteria were determined based on their reactions during the immunoagglutination serotyping with *Legionella* immune sera (Denka Seiken, Tokyo, Japan).

### 2.5. Cell Lines and Culture Conditions

HeLa cells were grown at 37°C and 5% CO_2_ in Dulbecco's modified Eagle's medium (DMEM, Sigma) containing 10% heat-inactivated fetal bovine serum (FBS, Biowest, Paris, France). A human monocytic cell line, THP-1 cells, was grown at 37°C and 5% CO_2_ in RPMI 1640 medium (Sigma), containing 10% heat-inactivated FBS. THP-1 cells were differentiated with 100 nM phorbol 12-myristate 13-acetate (PMA, Sigma) at 48 h prior to use. 

### 2.6. Intracellular Invasion and Growth Assays

Bacteria were added to a monolayer of HeLa cells or THP-1 cells in 48-well tissue culture dishes at multiplicity of infection (MOI) of 100 or 1, respectively. These plates were centrifuged for 5 min at 900 ×g and incubated for 1 h at 37°C. Extracellular bacteria were killed by gentamicin (50 *μ*g/mL) treatment for 1 h. To measure the invasion efficiency, cells were washed twice with phosphate-buffered saline (PBS) and lysed with cold distilled water. To measure the intracellular growth, the cells were incubated in fresh medium at 37°C for particular time and washed three times with PBS, followed by lysis with cold distilled water. Colony forming units (CFU) were determined by serial dilution on CYET. 

### 2.7. Cytotoxicity Measurement

Bacteria were added to a monolayer of HeLa cells or THP-1 cells in 48-well tissue culture dishes at MOI of 100 or 1, respectively. These plates were centrifuged for 5 min at 900 ×g and incubated for 1 h at 37°C. Extracellular bacteria were killed by gentamicin (50 *μ*g/mL) treatment for 1 h. Cells were washed twice with PBS and incubated in fresh medium at 37°C. At 24 or 48 h after incubation, the supernatants of infected cells were collected. Cytotoxicity was determined by measuring LDH release using a Cytotoxicity Detection Kit^PLUS^ (LDH) (Roche) according to the manufacturer's instructions. 

### 2.8. Statistical Analysis

Data are expressed as the mean of triplicate samples from three identically performed experiments, and the error bars represented the standard deviations. Statistical analyses were performed using Student's *t*-test. Statistically significant differences are indicated by asterisks (∗, *P* < 0.05). 

## 3. Results

### 3.1. Isolation and Identification

Twenty-two samples were collected from environmental water sites in Yamaguchi Prefecture, Japan. Samples were concentrated and spread on GVPC agar. Five possible colonies were obtained. Three were isolated from ashiyu foot spas, one was isolated from a water fountain, and the other was isolated from a pond. 

To confirm whether these isolates were *L. pneumophila* or not, the presence of *L. pneumophila* specific gene, *mip* [14], was tested by PCR. The *mip* gene was detected in all isolates, indicating that these isolates were *L. pneumophila*. We named these isolates Twr292, Ymt294, Ofk308, Ymg289, and Bnt314 (Tables 1 and 3).

The serotypes of these five isolates were then determined by immunoagglutination serotyping. Twr292, Ymt294, and Ymg289 were classified into serotype I, and Ofk308 and Bnt314 were classified into serotype IV (Table 3).

### 3.2. Growth in Liquid Medium

**Figure 1:**
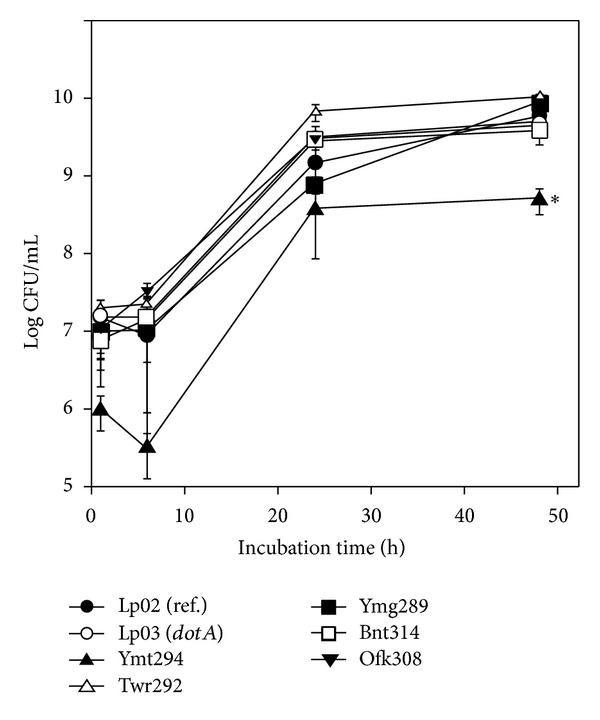
Growth of *L. pneumophila* isolates in liquid medium. Bacteria were grown in AYET. After 1, 24, and 48 h of incubation, samples were diluted with PBS and spread on CYET. All values represent the average and the standard deviation for three identical experiments. Statistically significant differences compared with the control are indicated by asterisks (∗, *P* < 0.05).

We compared the growth of the five isolates in AYET medium with that of the virulent reference strain Lp02 and the avirulent *ΔdotA* mutant Lp03, which lacks a functional Dot/Icm secretion system. Twr292, Ofk308, Ymg289, and Bnt314 showed comparable growth with Lp02 and Lp03. In contrast Ymt294 had shown lower growth rate. After 48 h, the number of Ymt294 was almost one-tenth of Lp02 and Lp03 (Figure 1). 

### 3.3. Invasion, Intracellular Growth, and Cytotoxicity in HeLa Cells

**Figure 2:**
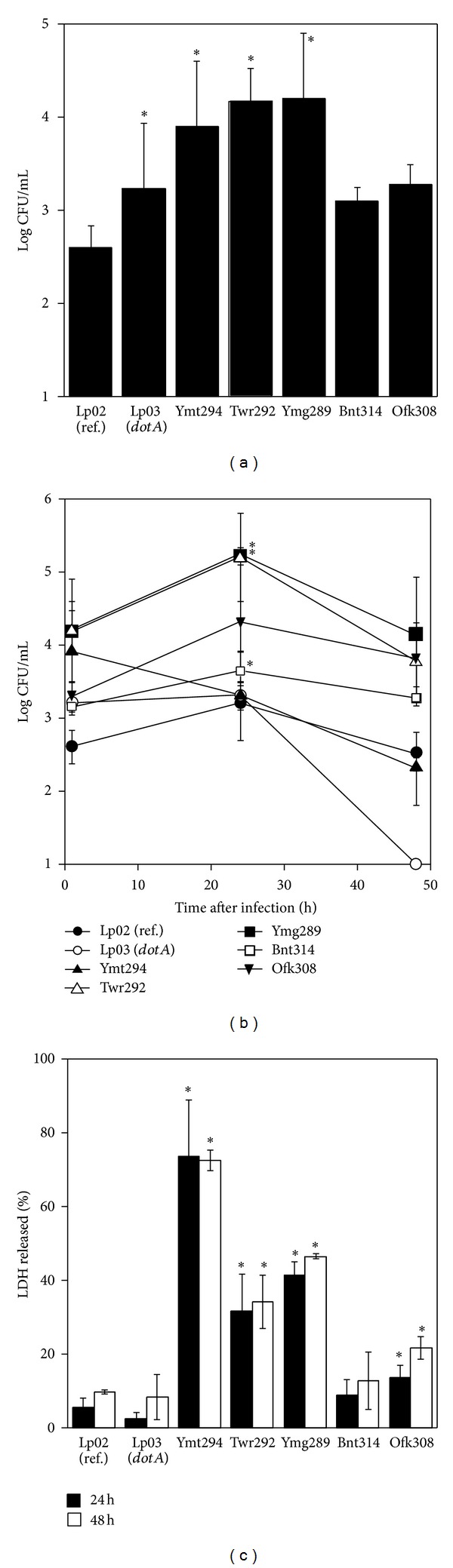
Invasion, intracellular growth, and cytotoxicity in HeLa cell. (a) HeLa cells were infected with *L. pneumophila* strains for 1 h. The infected cells were cultured in the presence of 50 *μ*g/mL gentamicin. After 1 h of incubation, the infected cells were washed with PBS and lysed with cold distilled water. CFU were determined by serial dilution on CYET. (b) HeLa cells were infected with *L. pneumophila* strains at MOI of 100 for 1 h. The infected cells were cultured in the presence of 50 *μ*g/mL gentamicin. The infected cells were cultured for 1, 24, and 48 h and washed with PBS followed by lysis with cold distilled water. CFU were determined by serial dilution on CYET. (c) HeLa cells were infected with *L. pneumophila* strains for 1 h. The infected cells were cultured in the presence of 50 *μ*g/mL gentamicin for 1 h. After 24 or 48 h incubation, the cells were washed and cultured in fresh medium. The supernatants of infected cells were collected, and the release of LDH was measured. All values represent the average and the standard deviation for three identical experiments. Statistically significant differences compared with the control are indicated by asterisks (∗, *P* < 0.05).

To investigate the intracellular behavior of the isolates, their invasion, growth, and cytotoxicity in HeLa cells were examined. HeLa cells were infected with the isolates, and the number of invaded *L. pneumophila* was counted at 1 h after infection. Ymt294, Twr292, and Ymg289 invaded HeLa cells more than ten times higher than reference strain Lp02 (Figure 2(a)). 

Intracellular growth of the isolates was measured by counting intracellular bacteria numbers at 24 and 48 h after infection. At 24 h after infection, Twr292, Ymg289, and Bnt314 showed higher growth and the bacterial number was more than ten times as compared with the reference strain Lp02. At 48 h after infection, the numbers of all isolates were decreased. The *ΔdotA* mutant Lp03 failed to replicate in HeLa cells, as previously reported [17] (Figure 2(b)).

The cytotoxicity of isolates was measured by LDH release assay and phase-contrast microscopy. At 24 and 48 h after infection, Ymt294, Twr292, and Ymg289 showed high cytotoxicity (Figure 2(c)). At 24 h after infection with isolates, cells were damaged and detached from the culture plates (Figures 4(a)–4(c) and data not shown).

### 3.4. Intracellular Growth and Cytotoxicity in THP-1 Cells

**Figure 3:**
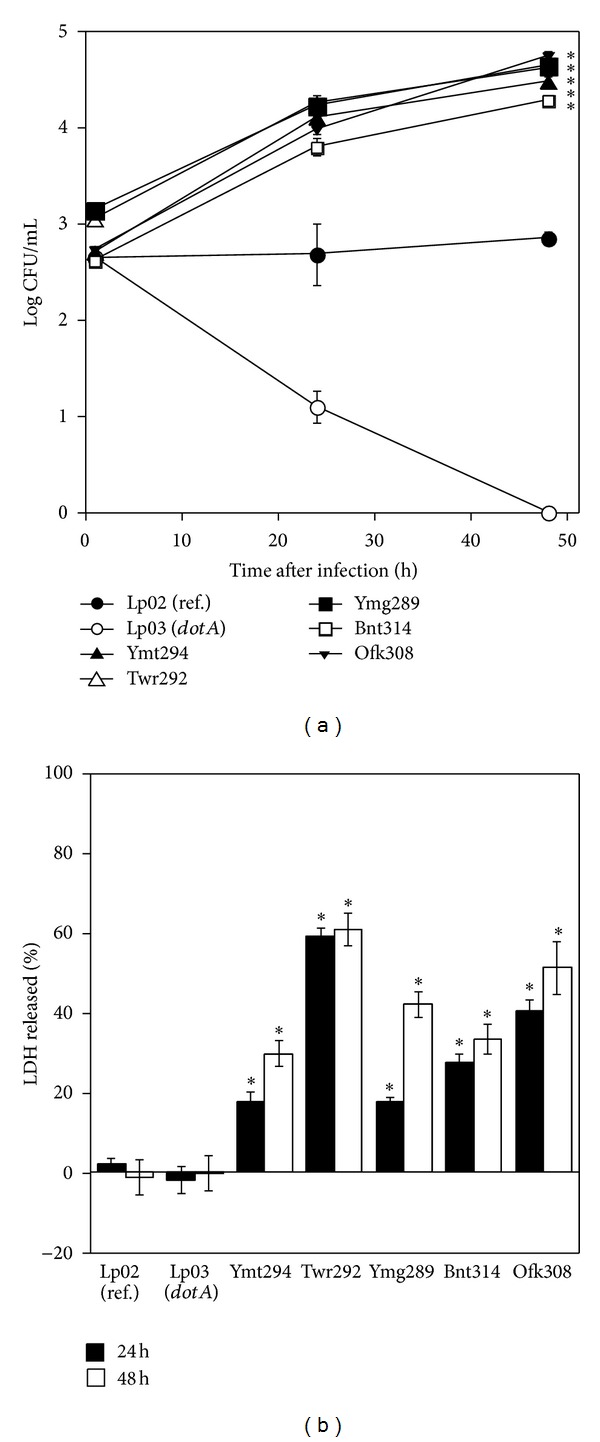
Intracellular growth and cytotoxicity in THP-1 cell. (a) THP-1 cells were infected with *L. pneumophila* strains at MOI of 1 for 1 h. The infected cells were cultured in the presence of 50 *μ*g/mL gentamicin. The infected cells were cultured for 1, 24, and 48 h and washed with PBS followed by lysis with cold distilled water. CFU were determined by serial dilution on CYET. (b) THP-1 cells were infected with *L. pneumophila* strains for 1 h. The infected cells were cultured in the presence of 50 *μ*g/mL gentamicin for 1 h. After 24 or 48 h of incubation, the cells were washed and cultured in fresh medium. The supernatants of infected cells were collected, and LDH release was measured. All values represent the average and the standard deviation for three identical experiments. Statistically significant differences compared with the control are indicated by asterisks (∗, *P* < 0.05).

**Figure 4:**
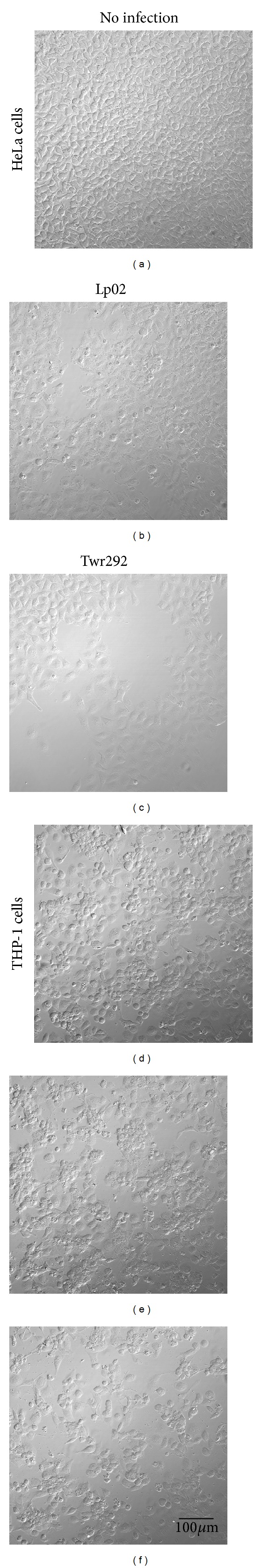
Cytotoxicity in HeLa and THP-1 cells. HeLa cells ((a)–(c)) and THP-1 cells ((d)-(e)) were infected with *L. pneumophila* strains Lp02 ((b) and (e)) or Twr292 ((c) and (f)) for 1 h. The infected cells were cultured in the presence of 50 *μ*g/mL gentamicin for 1 h. The cells were washed and cultured in fresh medium. After 24 h of incubation, the condition of cells was observed using phase-contrast microscope.

*L. pneumophila* resides predominantly in macrophages after infection; therefore, the growth and cytotoxicity of isolates were examined in a human macrophage cell line, THP-1 cells. At 24 h and 48 h after infection, all isolates showed potent growth. The numbers of these isolates were ten times higher than the reference strain Lp02. The *ΔdotA* mutant Lp03 failed to grow in THP-1 cells (Figure 3(a)). Moreover, all isolates showed higher cytotoxicity than the reference strain in THP-1 cells. Particularly Twr292 induced strong cytotoxicity (Figure 3(b)). Damaged and detached THP-1 cells were observed with phase-contrast microscopy after cells were infected with Twr292 (Figures 4(d)–4(f)).

### 3.5. Detection of Loci and Genes Related to Virulence Factor

To estimate whether these isolates are pathogenic to humans, the presence of genetic loci of *dot*, *lvh*, and *rtx* that encode typical virulence factors of *L. pneumophila* was examined. Loci of *dot* and *lvh* encode components of type IV secretion system that play an important role in intracellular growth [18]. Locus *rtx* encodes proteins involved in adherence, cytotoxicity, and pore formation [19]. The presence of *dot*, *lvh*, and *rtx* loci was tested by detecting *dotA*, *lvhB3*, and *rtxA* genes located in these loci, respectively, by PCR. The presence of the *hsp60* gene was also examined. *hsp60* encodes a 60 kDa heat shock protein (Hsp60) that enhances invasion and elicits cytokine expression in macrophages [20, 21]. These genes were detected in all five isolates (Table 4), indicating that these isolates are human pathogenic.

## 4. Discussion

In Japan, hot springs are reported to be the major infectious source for *L. pneumophila* [9–11]. However, there is little information about *L. pneumophila* in environmental waters other than hot springs. In this study, we tested 22 environmental water sites in Yamaguchi Prefecture, Japan. Eight were from ashiyu foot spas, seven were from water fountains, four were from basins, and three were from ponds. *L. pneumophila* was isolated from five sites (23%) (Table 1). Three were isolated from ashiyu foot spas (38%), one was isolated from a water fountain (14%), and the other was isolated from pond (33%). Interestingly, *L. pneumophila* was isolated mostly from ashiyu foot spas. Ashiyu foot spa is a type of hot spring where people bathe their feet. Ashiyu foot spa is usually in open air and freely available. Its temperature is generally controlled around 45°C. Older people often use this facility. For these people, *L. pneumophila*-containing aerosols generated from environmental waters could be a source of *L. pneumophila* infection. To the best of our knowledge, this is the first report related to isolation of *L. pneumophila* from ashiyu foot spa. Previous surveys of hot springs have demonstrated that around 30% of hot springs or public bathes were *L. pneumophila* positive [22, 23]. In this study, *L. pneumophila* was isolated from three of the eight sites (38%) of ashiyu foot spa sampled. These results may suggest an equivalent risk of contracting *L. pneumophila *at ashiyu foot spa as compared with hot spring. However, a more extensive survey is required to obtain more accurate epidemiological relevance and to analyze the risk of *L. pneumophila* infection from ashiyu foot spa. 

The growth of the *L. pneumophila* isolates in liquid medium was almost the same as reference strain Lp02, but Ymt294 showed lower growth rate (Figure 1). Since the number of Ymt294 was not increased from 24 to 48 h, the growth of Ymt294 seemed to be saturated at one-tenth of final concentration of other strains. Intracellular growth of these isolates was different in HeLa and THP-1 cells. In HeLa cells, growth of isolates was significantly higher than Lp02 at 24 h after infection. However, the numbers of intracellular bacteria were decreased at 48 h after infection (Figure 2(b)). Some isolates such as Ymt294, Twr292, and Ymg289 showed strong cytotoxicity in HeLa cells, and cells were detached from culture plate at 48 h (Figures 2(c) and 4). This detachment may be a dominant factor of decrease in intracellular growth of those isolates. In THP-1 cells, the numbers of intracellular bacteria were increased from 24 to 48 h, despite the high cytotoxicity of those isolates (Figures 3(a) and 3(b)). Consistent with the strong preference of *L. pneumophila* for macrophages, these results indicate that macrophages are more suitable for *L. pneumophila* growth than epithelial cells.

Since all isolates harbored genes of well-characterized virulence factors including *dot*, *lvh*,* rtx*, and *hsp60*, the relationship between virulence factors and cytotoxicity or intracellular growth was not clear. However, the existence of genes of the virulence factors may suggest that those isolates can be human pathogenic. In particular, the Twr292 isolate from ashiyu foot spa showed high intracellular growth and strong cytotoxicity in HeLa and THP-1 cells. In addition, the contamination level of Twr292 was very high (128 CFU/100 mL). According to the guidelines of Japan's Ministry of Health, Labour and Welfare, the concentration of *L. pneumophila* should be maintained below 10 CFU/100 mL in hot springs or public bathes. The concentration of Twr292 was more than ten times that of the defined standard. 

Overall, our results strongly suggest that ashiyu foot spa is a possible source of *L. pneumophila *infection. 

## Figures and Tables

**Table 1:** Detection of *Legionella pneumophila* from environmental waters.

Place	No. of collected points	No. of positive points	Positive rate (%)
Water fountain	7	1	14
Ashiyu foot spa	8	3	38
Basin	4	0	0
Pond	3	1	33

Total	22	5	23

**Table 2:** Oligonucleotides.

Name/region	Sequence (5′-3′)	Reference
lvh1/*lvhB3 *	attgggagcttctggcaata	This study
lvh2/*lvhB3 *	gctggggtgacctttgaata	This study
rtx1/*rtxA *	gctgcaaccacctctttgat	This study
rtx2/*rtxA *	caggggctggttatgttgat	This study
dot1/*dotA *	caaatccggcattcaaaatc	This study
dot2/*dotA *	ctattgtcgccttgggtgtt	This study
hsp1/*hsp60 *	gcgaatcgttgttaccaaagaaaac	[15]
hsp2/*hsp60 *	caatttgacgcattggagattcaatag	[15]
mip1/*mip *	ggtgactgcggctgttatgg	[16]
mip2/*mip *	ggccaataggtccgccaacg	[16]

**Table 3:** Isolation of *Legionella pneumophila* from PCR-positive sites.

Strain	Place	CFU/100 mL	Serotype
Ymg289	Water fountain	1	I
Twr292	Ashiyu foot spa	128	I
Ymt294	Ashiyu foot spa	2	I
Ofk308	Ashiyu foot spa	2	IV
Bnt314	Pond	4	IV

**Table 4:** Detection of loci and genes related to virulence factor.

Region	Lp02	Lp03	Ymg289	Twr292	Ymt294	Ofk308	Bnt314
*lvhB3 *	+	+	+	+	+	+	+
*rtxA *	+	+	+	+	+	+	+
*dotA *	+	−	+	+	+	+	+
*hsp60 *	+	+	+	+	+	+	+
